# Case study of pancreas-preserving enucleation in the treatment of isolated pancreatic metastases of renal cell carcinoma

**DOI:** 10.1186/1753-6561-9-S7-A23

**Published:** 2015-10-27

**Authors:** Toby D'cruz, JS Wong

**Affiliations:** 1 School of Medicine and Medical Sciences, University College Dublin, Dublin, Republic of Ireland; 2 Singapore General Hospital, Singapore

## Background

Pancreatic metastases are rare, accounting for 2.8% of cases in RCC, occurring as a result of haematogenous spread to the pancreas. High affinity of some renal cancer cells for the pancreatic parenchyma present solely as isolated pancreatic metastases.

Literature reviews have highlighted that aggressive surgery for isolated pancreatic metastasis has been shown to increase 5 year survival rates unto 60%; particularly nephrectomy & metastasectomy with adjuvant therapy.

The objective of this case study was to identify the benefits of pancreas-preserving enucleation in treatment of isolated pancreatic RCC metastases over traditional pancreatic resections.

## Report

Patient X was identified as a candidate for this case report.

Mr X. is a 77 year old fit and healthy male, presented with 2 day history of frank haematuria & weight loss of 3kg over 6 months with no associated fever, flank pain or dysuria. CT KUB revealed lobulated soft tissue density in the right kidney, suspicious for a tumour. Further staging investigations of CT TAP demonstrated a small enhancing nodule in pancreatic body confirmed as pancreatic metastasis with EUA FNA. The patient subsequently underwent Open Right Radical Nephrectomy with Enucleation of Pancreatic Metastasis.

## Discussion

Comparisons drawn between Pancreatic-sparing Enucleations and standard resection (Complete Pancreatectomy) explore variations in surgical challenges & post-operative complications. While there has been no difference in morbidity and recurrence noted compared to complete pancreatectomy; there has been significant reduction in post-operative diabetes mellitus.

## Conclusions

Pancreatic resections are associated with high rates of morbidity and mortality. A reduction in operative risk following pancreatic surgery have been demonstrated, in recent times. As such, Pancreas-sparing Enucleation & Enucleo-resection has been considered a worthy option. Preservation of pancreatic tissue allows for better quality of life without diabetes mellitus.

## Consent to publish

The patient had signed a written consent for publication of this abstract on Pancreatic Metastases.
